# Analysis of Urinary Prostate-Specific Antigen Glycoforms in Samples of Prostate Cancer and Benign Prostate Hyperplasia

**DOI:** 10.1155/2016/8915809

**Published:** 2016-03-15

**Authors:** Chun-Jen Hsiao, Tzong-Shin Tzai, Chein-Hung Chen, Wen-Horng Yang, Chung-Hsuan Chen

**Affiliations:** ^1^Genomics Research Center, Academia Sinica, Taipei 115, Taiwan; ^2^Institute of Microbiology and Immunology, National Yang-Ming University, Taipei 112, Taiwan; ^3^Department of Urology, National Chen Kung University Hospital, College of Medicine, National Chen Kung University, Tainan 701, Taiwan; ^4^Chemistry Department, National Taiwan University, Taipei 106, Taiwan; ^5^Institute of Atomic & Molecular Sciences, Academia Sinica, Taipei 106, Taiwan

## Abstract

Glycans of prostate-specific antigen (PSA) in prostate cancer were found to be different from that in benign disease. It is difficult to analyze heterogeneous PSA glycoforms in each individual specimen because of low protein abundance and the limitation of detection sensitivity. We developed a method for prostate cancer diagnosis based on PSA glycoforms. Specific glycoforms were screened in each clinical sample based on liquid chromatography-tandem mass spectrometry with ion accumulation. To look for potential biomarkers, normalized abundance of each glycoform in benign prostate hyperplasia (BPH) and in prostate cancer was evaluated. The PSA glycoform, Hex5HexNAc4NeuAc1dHex1, and monosialylated, sialylated, and unfucosylated glycoforms differed significantly between the prostate cancer and BPH samples. The detection sensitivity (87.5%) and specificity (60%) for prostate cancer identification are higher than those of the serum PSA marker. As low as 100 amol PSA could be detected with the ion accumulation method which has not been reported before. The improved detection specificity can help reduce unnecessary examinations.

## 1. Introduction

Prostate cancer is very common among men worldwide. Prostate-specific antigen (PSA) is a glycoprotein with an N-linked glycosylation site [1], and its level in serum is an FDA-approved prostate cancer marker [2–4]. However, the serum PSA concentration has a low diagnostic specificity for prostate cancer, which leads to many unnecessary patient biopsies [5]. Therefore, there is an urgent need for markers with better specificity. The alterations of protein glycoforms is one of the candidates.

To analyze PSA glycoforms, mass spectrometry (MS) [6–10] has been adopted. Glycan composition, protein binding sites, and peptide backbone sequences can be detected by tandem MS. However, glycoforms identification is hampered by glycan heterogeneity. Liquid chromatography (LC), which is often coupled with mass spectrometry, can be used to separate compounds from a mixture to improve the detection sensitivity. In addition, when the MS is set to detect a specific ion with a known mass-to-charge ratio (*m/z*), more ions can be accumulated in a trap for MS analysis. In a typical LC-MS analysis, only a small portion of the sample under the specific LC peak is used for mass spectrometry. A typical LC peak lasts from seconds to minutes, but a typical round of ion trapping lasts from only a few milliseconds to a few hundred milliseconds. Therefore, most of the samples eluted from the LC are not used for MS analysis. Because the quantity of each specific PSA glycoform can be very low, ion accumulation can improve the detection sensitivity by orders of magnitude. Using this ion accumulation method, the detection sensitivity can be further improved.

Glycopeptide quantification is a challenge because of the lack of synthetic standards to establish calibration curves. Label-free approaches have been developed for peptide glycoform analysis [11–13]. In LC-MS, the area under an LC peak represents the abundance of the selected molecules. This method has been used to evaluate the specific posttranslational modifications of a protein [14, 15]. To avoid interference from sample processing, the glycopeptide abundance can be evaluated by normalizing to an internal reference peptide from the same protein. The reduced interference from sample processing means that this approach can be used to investigate the relative abundance of each PSA glycoform in an individual clinical sample. Using this approach, the relative abundance of each specific glycoform of haptoglobin and of immunoglobulin G has been accurately measured [11–13]. Nevertheless, no studies have been reported for each PSA glycoform by screening clinical samples using LC-MS.

Recently, PSA glycan profiles have been studied as potential prostate cancer markers [6–8, 16–21]. However, detection of multiple PSA glycoforms in each individual sample was seldom pursued because of the low PSA levels in the samples and because of the heterogeneity of the PSA glycoforms, making the detection of each specific glycoform much more difficult. Urine contains more PSA than serum [22], and it is easier to obtain patients' permissions to collect it. Therefore, each individual sample can be analyzed without the need for pooling. As in semen, urinary PSA exists in its free form [22–24]. It is beneficial to pursue analysis of glycan by measuring glycopeptide. In addition, urinary PSA is directly secreted from the prostate to the urine; unlike serum PSA, it is not renally filtered [24]. Therefore, the PSA composition of the urine should be closer to that of the prostate gland. Urine could be a better specimen than serum for PSA glycosylation analysis in prostate cancer diagnosis.

In this study, we evaluated the relative abundance of each urinary PSA glycoform in clinical samples. Specific glycopeptides and a specific PSA peptide that serves as internal reference were detected using LC-MS. The degree of PSA glycosylation was evaluated by calculating the ratio of the abundance of each glycopeptide to that of the PSA internal reference peptide. Specific PSA glycoforms in clinical samples were identified by ion accumulation method. The analysis of the clinical urine samples showed that the relative abundance of several glycoforms differed between BPH and prostate cancer. Therefore, these glycoforms could be potential biomarkers to distinguish between BPH and prostate cancer.

## 2. Materials and Methods

To quantify the site-specific PSA glycopeptides in clinical samples, the following processes were performed: human seminal PSA was used to investigate the glycopeptide backbone sequence and to select the internal reference peptide. The peptide backbone sequence of the PSA glycopeptides was identified, and the MS2 profile of each glycoform was obtained. Next, the PSA glycopeptide distributions in pooled BPH and PCa urinary samples were identified. Then, for each individual clinical sample, the ratios of these specific PSA glycopeptides to the reference peptide were determined. Potential glycoform markers that are differentially expressed between BPH and PCa could be discovered.

### 2.1. Materials and Reagents

Human seminal PSA (P3338, ≥95% purity in SDS-PAGE) and iodoacetamide (IAA, I6125) were purchased from Sigma-Aldrich (St. Louis, MO, USA). Human PSA monoclonal antibodies were purchased from R&D Systems, Inc. (MAB1344, Minneapolis, MN, USA). Dynabeads® Protein G was purchased from Life Technologies (10003, Waltham, MA, USA). Centrifugal filter tubes were purchased from Millipore (Amicon® Ultra-15 centrifugal filter units, 10 K NMWL, Billerica, MA, USA). Chymotrypsin was purchased from Promega (V1062, Madison, WI, USA). The deglycosylation enzyme PNGase F was purchased from NEB (P0704, New England Biolabs, ON, Canada). DTT (1,4-dithiothreitol, 111474) was purchased from Merck (Darmstadt, Germany). The C18 nanospray column (75 *µ*m × 20 cm, 2.5 *µ*m particle size) was packed in-house. The C18 particles were purchased from Dr. Maisch HPLC GmbH (ReproSil-Pur Basic®, 2.5 *µ*m, Ammerbuch-Entringen, Germany).

### 2.2. Clinical Samples

Urine samples were collected from the National Cheng Kung University Hospital (Tainan, Taiwan). In total, 61 BPH samples and 38 prostate cancer samples were tested in this study. Clinical information and urine specimens were collected with the approval of the Institutional Review Board (IRB) of Academia Sinica (Taipei, Taiwan) and the National Cheng Kung University Hospital. Clinical information such as serum PSA level and ages of patients is shown in Table 1. There are no significant differences of serum PSA level between the patients of BPH and prostate cancer in this study.

Urine (50–100 mL per patient) was collected then stored at −20°C before processing. Before being used in experiments, the urine sample was thawed at room temperature and then centrifuged (1500 g, 10 min) to remove cell debris and precipitates. The total protein in the supernatant of 50 mL of urine was concentrated using Amicon Ultra-15 centrifugal filter units (5000 g, 45 min/run at 4°C) and was then ready to be immunoprecipitated. Ten microliters of each concentrated urine sample was pooled to generate the BPH and PCa sample pools.

### 2.3. Urinary PSA Immunoprecipitation and In-Gel Digestion

The anti-PSA monoclonal antibody (10 *µ*g) was dissolved in 200 *µ*L PBST (phosphate buffered saline plus 0.05% Tween-20) and incubated with protein G magnetic beads (1.5 mg) at room temperature for 30 min. After three washes with PBST, the antibody-coupled beads were incubated with concentrated urine for 1 hour at room temperature to capture the PSA. The protein on the beads was denatured in 20 *µ*L SDS sample buffer by boiling at 95°C for 5 min and then separated by SDS-PAGE (12% separating gel). The gel was then stained with Coomassie Brilliant Blue. Seminal PSA (2 *µ*g) was captured using anti-PSA beads and then denatured by boiling in SDS sample buffer (20 *µ*L) at 95°C for 5 min. The denatured protein was subjected to SDS-PAGE separation followed by in-gel digestion.

In-gel protein digestion was carried out as described previously [25]. Briefly, the stained protein band at 28–30 kDa was removed and cut into small pieces (roughly 1 mm^2^ squares). These pieces were then destained in 40% acetonitrile (ACN) for 10 min, reduced in 10 mM DTT at 56°C for 60 min, alkylated in 55 mM IAA at room temperature for 45 min in the dark, and then dehydrated in 100% ACN. Then, 100 ng of chymotrypsin dissolved in 40 *µ*L of 50 mM ammonium bicarbonate was added to the dehydrated gel pieces and incubated at 37°C for 18 hours. The digested peptides were eluted twice from the gel pieces using 50 *µ*L of 60% ACN/1% trifluoroacetic acid. The collected products were vacuum dried and then reconstituted in 20 *µ*L of deionized water for MS analysis.

### 2.4. Mass Spectrometry

LC-MS was used to study the seminal and pooled urinary PSA samples. The peptides and N-glycopeptides generated from the chymotrypsin digestion were analyzed with an LTQ-Orbitrap XL Mass Spectrometer (Thermo Scientific, San Jose, CA, USA) equipped with a nanoelectrospray ion source, an Agilent 1100 Series binary high-performance liquid chromatography pump (Agilent Technologies, Palo Alto, CA, USA), and a FAMOS autosampler (LC Packing, San Francisco, CA, USA). A total of 5 *µ*L of samples were injected into a manually packed precolumn (150 *µ*m ID × 30 mm, 5 *µ*m, 200 Å) at a 10 *µ*L/min flow rate. Chromatographic separation was performed over 90 min on a manually packed reversed phase C18 nanocolumn (75 *µ*m ID × 200 mm, 3 *µ*m, 200 Å) using 0.1% formic acid in water as mobile phase A, 0.1% formic acid in 80% acetonitrile as mobile phase B, and a split flow rate of 300 nL/min. The full-scan mass rage was set from* m/z* 320 to 2000 with 60,000 resolution at* m/z* = 400. The top ten most intense ions were sequentially isolated for MS2 by LTQ. The electrospray voltage was maintained at 1.8 kV, and the capillary temperature was set to 200°C. The glycopeptides were detected based on the mass and were confirmed using the MS2 spectra of oxonium ions and of peptide ions with a core N-acetylhexosamine (HexNAc) (Y1 ions) [26, 27]. To confirm the peptide backbone sequences, Y1 ions were captured and fragmented with a Velos Pro to produce MS3 spectra. Full MS/MS scans (*m/z* 100–2000) of high energy C-trap dissociation (HCD) were pursued. The minimum required signal intensity was set at 10,000 count/sec, and the isolation width of the precursor ion was set at 10 Da. The default activation time was 2 ms, and the normalized collision energy (NCE) for fragmentation was 28%. MS3 fragmentation was achieved in CID mode with a minimum signal intensity of 100 counts/sec, the isolation width of the precursor ion was set to 20 Da, the NCE for fragmentation was set to 35%, and the activation time was set to 10 ms. Y1 ion peptide sequence was obtained using the Mascot search engine for mass spectra and was used to identify the glycopeptide backbone sequence.

To analyze the selected ions in the individual sample, a 7-Tesla LTQ-FT Ultra Mass Spectrometer (linear quadrupole ion trap Fourier transform ion cyclotron resonance, Thermo Scientific, San Jose, CA, USA) equipped with nanoelectrospray ionization was used, and the total LC running time was 30 min. The full-scan MS spectra (*m/z* 320–1600) were acquired using the FTICR with a mass resolution of 200,000 at* m/z* = 400. Ions of the specific glycopeptides and the internal reference peptide were trapped with ion accumulation time of 250 ms, the NCE for fragmentation was set at 35%, and the isolation width of the precursor ions was set to 3 Da.

For samples that were difficult to detect due to low quantities of PSA, the internal reference peptide ([M + 2H]^2+^ = 485.78) was selected and analyzed via the ion accumulation method using a Velos Pro with CID fragmentation and elution from a C18 nanoelectrospray column. The maximum ion accumulation time was set to 3000 ms, and the isolation width of the precursor ions was set to 2 Da.

### 2.5. Database Search of MS/MS Spectra

The MS/MS data were processed and searched using the Mascot search engine (Matrix Science, Boston, MA, USA). The peak-list files were obtained from the MS/MS data using Extract_msm 5.0 software (Thermo Scientific) and they included the mass values, the intensity, and the charge of the precursor ions (parent ions with +2 or +3 charges in this study). The search parameters used in this study were IPI_Human v. 3.74 as the database, chymotrypsin as the enzyme, up to 2 missed cleavages, a peptide ion mass matching tolerance of 10 ppm, and a fragment ion mass tolerance of 0.8 Da. Oxidation (M) and N-terminal protein acetylation were set as variable modifications, and carbamidomethyl (C) was set as a fixed modification.

### 2.6. Identification of PSA Glycopeptides

Glycopeptide identification was carried out using software developed in-house. Putative glycopeptides were listed as the molecular weight of the deconvoluted LTQ-Orbitrap XL mass spectra and were matched against an established glycopeptide database. The listed glycopeptides were confirmed by MS2 of the oxonium ions and Y1 ions.

### 2.7. Quantitation of PSA Glycosylation

Each PSA glycopeptide and the internal reference peptide were quantified with a label-free method that used the peak area of MS1 under the LC curve, and the peak area was calculated using Xcalibur Software (Thermo Scientific). The selected PSA glycopeptides were eluted with a retention time of ±1.5 min within a 30 min LC run. The MS1 peaks of each glycopeptide and of the reference peptide were obtained with error tolerance settings of 500 ppm and 10 ppm, respectively. All selected glycopeptides were validated via MS2 spectra. The normalized abundance of each PSA glycoform was calculated according to the following equation:

(1) Level  of  PSA  glycopeptide=Glycopeptide  ion  abundancePSA  protein  internal  reference  peptide  ion  abundance×100.

### 2.8. Statistical Analysis

To evaluate the differences in normalized glycopeptide abundance in the clinical samples, two-tailed *t*-tests were used. Receiver operating characteristic (ROC) analysis and the area under the ROC curve (AUC) were used to evaluate sensitivity, specificity, and performance under optimal conditions. The graph was created by plotting the sensitivity and the false positive rate at various thresholds. AUC is a tool that has been used to describe the discriminatory ability of a test under various thresholds. Student's *t*-tests, ROC, and AUC were calculated using GraphPad Prism 5 software. The cut-off value was determined by calculating the Youden Index (*J*), which defines the maximum potential effectiveness of a biomarker. The Youden Index was calculated as the maximum value of “Sensitivity + Specificity − 1.”

## 3. Results

We developed a method for the quantitation of urinary PSA and its glycoforms based on relative abundance. Purified seminal PSA was used as a standard for studying the correlation of PSA quantity and normalized glycoforms abundance. The method was then applied to pool BPH and PCa urine samples from each individual. Because some glycopeptides were low abundance in some samples, specific molecules were ion accumulated for MS2 to increase detection sensitivity. Using this strategy, the abundance of PSA and the degree of PSA glycosylation was monitored simultaneously.

### 3.1. Quantitation of PSA Protein and Glycopeptides

**Figure 1:**
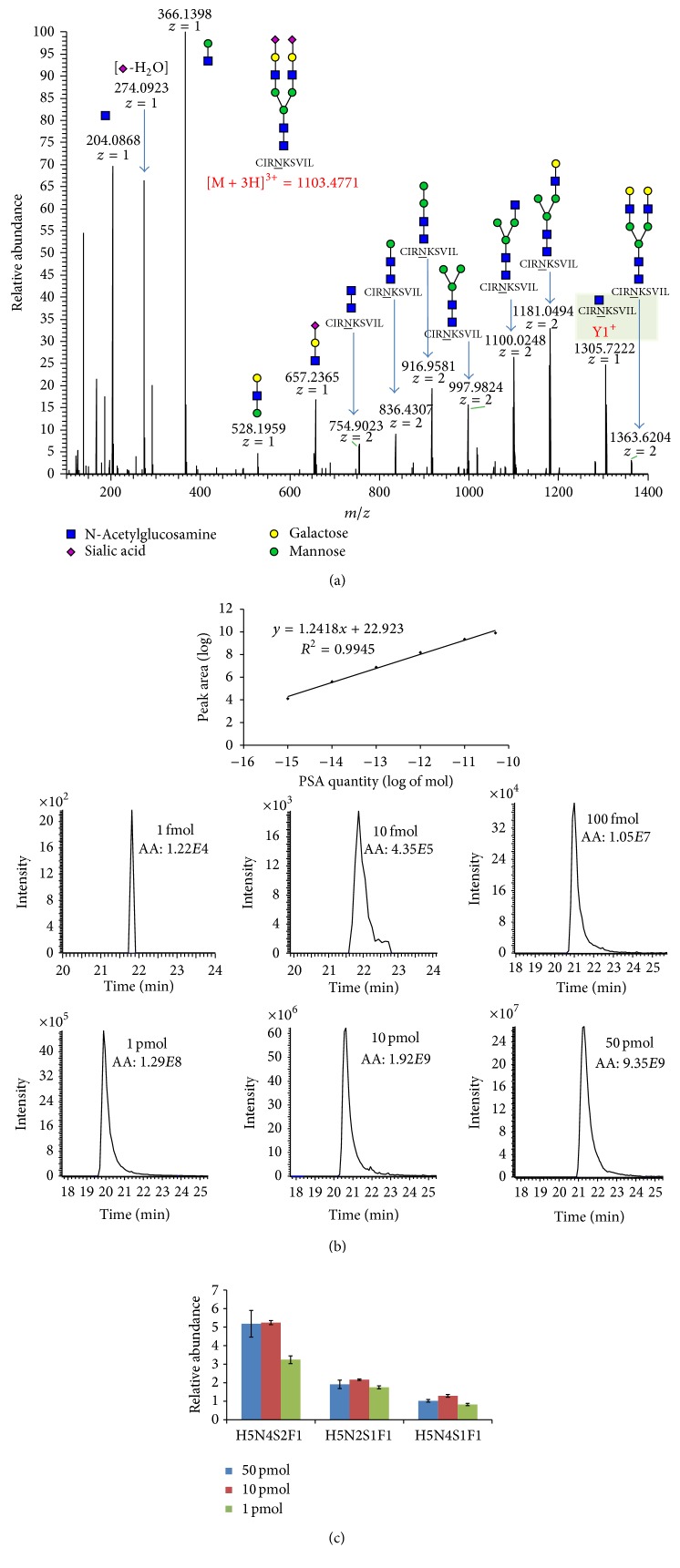
Identification and quantitation of PSA glycopeptide via tandem MS. (a) The MS2 of the CIRNKSVIL-Hex5HexNAc4NeuAc2 glycopeptide was identified. Oxonium ions, glycopeptide fragment ions with oligosaccharide fragments, and Y1 ion were all identified. The monosaccharide symbols are N-acetylglucosamine (blue square); mannose (green circle); galactose (yellow circle); and sialic acid (purpule diamond). (b) The dynamic range of the selected PSA internal reference peptide. Each spot is the average of triplicate. The inlet is MS1 peak of the reference peptide detected in varied PSA quantity. Peak intensity and the area under the peak (AA) are shown. (c) The normalized abundance of specific PSA glycopeptides in varying protein quantities. The error bars represent the standard deviations of triplicate results. The glycoform abbreviations are as follows: hexose (H), N-acetyl hexose (N), N-acetylneuraminic acid (sialic acid, S), and fucose (deoxyhexose, F).

Human seminal PSA was used as a standard to establish the method. The peptides generated by chymotrypsin digestion were analyzed with an LC-LTQ-Orbitrap XL and were searched using Mascot. Glycopeptides were detected based on molecular weight (Table 1) and validated by MS2 fragments with oxonium ions and glycosidic peptide ions (Figure 1(a)). Peptide backbone sequence was validated by deglycosylation and MS3 of Y1 ion.

To evaluate the relative abundance of the different PSA glycopeptides, an internal reference peptide was selected for normalization based on several criteria. First, the peptide needed to be unique to the PSA protein. The selected peptide had to be consistently detectable, and its abundance should be proportional to the PSA level. The dynamic range of the peptide needed to be large enough to evaluate the protein level. Of the chymotrypsin-digested PSA peptides, a peptide that fits the above criteria, IKDTIVANP ([M + 2H]^2+^ = 485.7823), was identified. This peptide is located at the C-terminus of PSA and has a sequence that is unique to PSA; its sequence does not overlap with those of other peptides. The peptide's signal was also consistently higher than that of other PSA peptides. The abundance of the peptide (the peak MS1 area under LC curve) was proportional to the quantity of PSA protein (Figure 1(b)). The dynamic range was 1 fmol to 10 pmol (corresponding to 0.3 pg and 300 ng of PSA protein). This peptide was selected as the internal reference peptide for the normalization of glycopeptide abundance.

The signal of the glycopeptides obtained from mass spectrometry is generally lower than that of the peptides without linked glycans [12, 28] which is consistent with our observations. The detection limit for the abundant glycopeptides was approximately 100 fmol. When the PSA protein quantity was lower than 100 fmol, each specific glycopeptide was difficult to detect. An analysis of the abundance of specific glycopeptides relative to the internal reference peptide showed that their abundance was proportional to the overall quantity of PSA (Figure 1(c)). Therefore, specific PSA glycoforms can be evaluated using their normalized abundance.

A total of 23 glycoforms were identified from the seminal PSA (Table 2), most of which were biantennary glycans. No tri- or tetra-antennary glycans were identified. High-mannose glycans were found with low abundance. The most abundant glycoform was Hex5HexNAc4NeuAc2dHex1 (H5N4S2F1), which made up more than 25% of all identified glycoforms. These observed seminal PSA glycoforms were consistent with previous studies [9, 10, 29].

### 3.2. Detection of Urinary PSA Glycopeptides in Pooled Clinical Samples

Urinary PSA glycopeptides from pooled BPH and PCa samples were analyzed with an LTQ-Orbitrap XL, which provided a high-accuracy* m/z*. The glycopeptides and the internal reference peptide were eluted at different times using LC with a run time of 90 minutes. Based on their molecular weights and elution times, eleven glycoforms were identified from both sample types. Eight PSA glycoforms were confirmed with MS2 spectra (Table 3). High-mannose and complex glycoforms were detected, and H5N4S2F1 was the most abundant PSA glycoform. The normalized abundance of each glycoform varied between the pooled BPH and PCa samples, and Hex4HexNAc2 (H4N2) was only detected in the BPH sample, not the PCa sample. However, the differences in the pooled samples cannot reflect the diversity of the individual samples. Therefore, the glycoforms were also investigated in each individual sample.

### 3.3. Glycoform Validation in Individual Clinical Samples

To investigate glycoforms in each clinical sample, each specific glycopeptide and the reference peptide were selected for MS analysis (Table 4). We focused on these selected ions and screened their distribution in all clinical samples using an LTQ-FT Ultra MS. In total, 61 BPH and 38 prostate cancer samples were analyzed for the six selected glycopeptides and the internal reference peptide using LC with a run time of 30 min.

The PSA reference peptide could be detected in 59 BPH and 34 PCa samples. Among these samples, at least one selected glycopeptide in 43 BPH and 20 PCa samples was detected (Table 4). For samples that contained at least 100 fmol of PSA one or more of the selected glycopeptides were expected to be detected. No significant differences of serum PSA level between BPH and prostate cancer. Reference peptide but not selected glycopeptides was detected in 14 BPH and 10 PCa samples. Immunoprecipitated urinary PSA was extremely low and difficult to be observed on the gel. However, PSA in these samples was validated by western blot. The reason of glycopeptides undetectable was due to sample variations instead of experimental problems. According to the results, none of the selected glycopeptides were BPH- or PCa-specific in any sample. Among the 6 glycoforms, H5N4S1F1 was the most commonly observed in BPH samples (42/59, 71%), and H6N3S1 was the second most common (18/33, 69%). In the PCa samples, H5N4S2F1 and H6N3S1 were the major isoforms observed (55%). The relative abundance of each glycoform in each sample was estimated (Table 4). Sialylation and fucosylation of PSA glycans were correlated to prostate cancer [19]. These glycan categories were evaluated as grouped glycoforms to sum the level of specific selected glycopeptides. Unsialylated, monosialylated, desialyalted, sialylated, fucosylated, unfucosylated, and all selected glycoforms combined (total) in each individual sample were evaluated, respectively. Among the six selected glycoforms, the normalized relative abundance of H5N4S1F1 and H6N3S1 showed significant differences between BPH and PCa. For grouped glycoforms, monosialylated, unfucosylated, and total showed different expression levels (*p* < 0.05) (Table 4). However, there are no significant differences between BPH and prostate cancer samples in serum PSA level.

The ROC curve and the AUC of the H5N4S1F1 glycoforms showed significant differences and moderate discrimination power (Table 5). Below the cut-off value, the prostate cancer detection sensitivity is 93%, and the specificity is 59%. The sensitivity is defined as the number of true positives divided by the number of all verified positives. The specificity is defined as the number of true negatives divided by the number of all verified negatives. The AUC of the grouped glycoforms was analyzed based on the samples which was able to detect the grouped glycoforms. For example, grouped monosialylated glycoform was detected in 43 BPH and 19 PCa. The AUC of grouped monosialylated glycoform was analyzed by these 62 samples. By the same way, the AUC of grouped total was analyzed by the results of 43 BPH and 20 PCa. Because the sample number varied between different grouped glycoforms, different groups were considered, respectively. The sialylated, monosialylated, and unfucosylated glycoforms showed lower discrimination power (AUC < 0.7) than that of all groups combined (0.7 < AUC < 0.8) (Table 5). The monosialylated group showed high sensitivity (100%) and weak specificity (47.5%) for prostate cancer detection. The prostate cancer detection sensitivity of all glycoforms combined (total) was 87.5% and the specificity was 60%. Both H5N4S1F1 and the “total” group differed significantly between BPH and PCa.

### 3.4. Further Exploration of Samples without Enough PSA for Routine Analysis

The internal reference peptide for PSA could not be detected in six samples with a routine analysis. To confirm the presence of PSA, the specific peptide was detected with the ion accumulation method using a Velos Pro, which traps a specific* m/z* and consequently increases sensitivity. The maximum ion accumulation time can be extended from 250 ms to 3000 ms. In principle, the sensitivity could be improved more than 10-fold. Therefore, we focused on analyzing the PSA internal reference peptide using the Velos Pro.

PSA was not able to be detected in 2 BPH samples (BPH066 and BPH069) and 4 prostate cancer samples (PCa010, PCa018, PCa029, and PCa030) by ELISA. However, the internal reference peptide was detected in all 6 samples via validation of its characteristic fragment ions (Table 6). Using the ion accumulation method, the detection limit of the protein peptide fragments was less than 100 amol, which is far lower than the ELISA detection limit of 1 ng/mL.

## 4. Discussion

An interlaboratory study of human seminal PSA glycosylation was published in 2013 [9], and two of the participating laboratories showed detailed results using bottom-up and top-down approaches [10, 29]. In these studies, major and intermediate glycoforms were detected, and these glycoforms represented more than 80% of the total glycoform content. These abundant glycoforms were also identified in our report on seminal PSA. However, their presence was not validated in clinical serum or urinary PSA samples. In addition, the abundance of each glycoform was estimated based on the relative intensity of each glycan compared with all discovered glycoforms. Depending on the number of discovered PSA glycans, this value varies between experiments. In this work, we not only identify and evaluate these abundant glycoforms in seminal PSA but also analyze each glycoform in clinical urine samples. Highly purified PSA is not necessary for the assay. Furthermore, the normalized abundance of each glycopeptide can provide a standard method to evaluate the abundance of each glycopeptide across different samples. This strategy can also be applied to the analysis of glycoform abundance for other glycoproteins.

The reference peptide but not the selected glycopeptides in some clinical samples was detected. It was due to biological variance or the limitation of sample collection. First, the glycopeptides we screened here were too low to be detected. Some other glycoforms may exist but too low to be detected. To extend the screen list of glycoforms may be helpful. Second, urinary PSA levels in these samples were extremely low. Glycopeptides were difficult to detect in the samples with PSA protein lower than 100 fmole. Collecting higher amounts of urinary PSA, such as first void urine in the morning, would improve the results. As long as the selected glycoforms could be detected, the possibility of prostate cancer could be evaluated.

It is easier to collect large volumes of urine than collect large volumes of serum. However, the quantity of PSA in urine samples varies widely depending on the collection time [30, 31]. The first 5–10 mL of urine in the first morning void contains more PSA [30], whereas randomly collected urine samples and samples collected from midstream contain less PSA. Although more urinary PSA can be collected after prostate massage, this process is inconvenient and uncomfortable for patients. The urine specimens used in this study were collected randomly, potentially leading to low PSA levels. Therefore, the absolute quantity of each glycopeptide in the sample may not provide truly accurate information for disease diagnosis. However, the glycopeptide abundance relative to that of the internal reference peptide can reduce the effects of sample collection and sample processing [12]. In this study, we could evaluate each PSA glycopeptide in samples containing as low as 100 fmol of PSA. To the best of our knowledge, this level of sensitivity has not been reported before.

Although the detection of PSA glycoforms has been published in the past, no reports have measured specific glycoforms in individual samples. Investigating the glycoform distribution in each specimen is extremely difficult. Some glycoforms may be detected in certain samples, but the same glycoforms might not be detected in a pooled sample due to their dilution. For example, glycoform H4N2 was not detectable in the pooled PCa sample but could be detected in six PCa samples. This observation indicates that some low-abundance glycopeptides cannot be efficiently detected by pooling samples. Therefore, the method used here is an improvement because it can screen for the distribution of multiple glycoforms in clinical specimens.

According to the report from the American Cancer Society, the use of serum PSA as a prostate cancer biomarker has a sensitivity and specificity of 63% and 35%, respectively [4]. This low specificity could lead to an incorrect diagnosis and unnecessary treatment for certain patients [5]. In 65% to 75% of patients with elevated PSA, biopsies showed no cancer [32–34]. In these cases, the elevated serum PSA may instead be due to bacterial prostatitis and acute urinary retention [35]. There is a need to develop a highly specific diagnostic approach for prostate cancer. Ideally, the use of PSA testing as a marker would be individually tailored [36]. However, current reports on PSA glycoform analysis are not suitable for this purpose. In this study, multiple glycoforms were simultaneously screened in each sample. In addition, no significant differences of serum PSA level between BPH and PCa patients are analyzed in this study. However, these patients could be differentiated by evaluating the level of PSA glycoforms (H4N5S1F1) or groups of glycoforms with good specificity (60%). This greater specificity may improve prostate cancer diagnosis and reduce unnecessary biopsies. Because no specific glycoform could be detected in every sample, it is reasonable to combine several glycoforms to evaluate the glycan distribution among the samples. Although a large-scale study is needed to reach a definite conclusion, the urinary PSA glycoforms reported here could be a potential choice for prostate cancer diagnosis.

The ion accumulation approach can provide a more sensitive method to detect low levels of PSA (<1 fmol). A similar approach could be applied to study other rare species of known molecular weight in a sample. This approach should be valuable in glycomic and glycoproteomic analyses to find low-abundance biomarkers.

## 5. Conclusion

We analyzed the urinary PSA glycoforms in individual clinical samples and found significant differences in individual glycoforms and in several groups of glycoforms. Label-free quantitation relative to an internal reference peptide simplifies the search for potential markers. This is the first report to screen for specific PSA glycoforms in each individual clinical sample. The method could be used for large-scale studies that investigate glycoform markers. Compared with other specimens, urine provides a noninvasive choice for prostate cancer diagnosis. In addition, a highly sensitive ion accumulation approach using specific ions can be used to detect low-abundance glycoproteins in clinical samples.

## Figures and Tables

**Table 1:** Patients information collected in this study.

	BPH	PCa	*p* value
Samples	61	38	
Age	48~84	56~82	
Mean ± SD	66.5	71.6	
Median	67	73	
Serum PSA (ng/mL)	0.73~78.0	6.19~27354	0.247^#^
Mean ± SD	13.44 ± 14.42	857.93 ± 4430.24	
Median	9.39	26.67	
Gleason score		6~10	

^#^No significant differences.

**Table 2:** Identified human seminal PSA glycopeptides.

[M + 3H]^3+^ ^(a)^	Glycan composition^(b)^	Abundance	
		Peak area	Normalized^(c)^
665.6575	H3N2	3.06*E* + 06	0.51
720.0094	H4N2	9.44*E* + 06	1.57
773.6926	H5N2	2.20*E* + 07	3.67
836.0542	H4N3F1	5.91*E* + 06	0.98
884.4000	H4N3S1	1.10*E* + 07	1.84
895.4034	H6N3	5.01*E* + 06	0.83
898.0755	H3N4S1	4.10*E* + 06	0.68
933.0860	H4N3S1F1	2.26*E* + 07	3.77
946.7615	H3N4S1F1	5.84*E* + 06	0.97
952.0931	H4N4S1	5.41*E* + 06	0.90
957.7649	H5N4F1	5.80*E* + 06	0.96
992.4352	H6N3S1	4.24*E* + 07	7.06
1000.7791	H4N4S1F1	1.10*E* + 07	1.83
1006.1107	H5N4S1	1.12*E* + 07	1.87
1014.4546	H3N5S1F1	7.59*E* + 06	1.26
1019.7863	H4N5S1	4.20*E* + 06	0.70
1054.7967	H5N4S1F1	1.92*E* + 07	3.20
1068.4722	H4N5S1F1	7.81*E* + 06	1.30
1103.1425	H5N4S2	2.80*E* + 07	4.65
1116.8181	H4N5S2	1.20*E* + 07	1.99
1151.8285	H5N4S2F1	6.94*E* + 07	11.54
1157.1601	H6N4S2	9.35*E* + 06	1.56
1165.5040	H4N5S2F1	2.01*E* + 07	3.34

(a) Monoisotopic masses are given throughout.

(b) The abbreviations of glycans are shown: H: hexose (Hex); N: N-acetylhexosamine (HexNAc); S: sialic acid (NeuAc); F: fucose (dHex)

(c) Normalized means the normalized abundance, which is the level of PSA glycosylation.

**Table 3:** The urinary PSA glycoforms were identified in pooled samples.

Glycan composition	Normalized abundance^*∗*^	
	BPH	PCa
H4N2	0.52	ND^*∗∗*^
H5N2	0.42	0.59
H6N3	0.18	0.08
H6N3S1	0.80	1.60
H5N4S1F1	1.02	0.77
H4N5S1F1	0.23	0.16
H5N4S2	0.23	0.43
H5N4S2F1	1.45	2.51

^*∗*^The results were the average of two measurements.

^*∗∗*^ND: not detected.

**Table 4:** The normalized abundance of each selected and grouped glycoform detected in each individual sample.

	Selected glycoforms						Grouped glycoforms						
	H4N2	H5N4S2F1	H5N4S2	H5N4S1F1	H6N3S1	H5N2	Unsialylated	Monosialylated	Disialylated	Sialylated	Fucosylated	Unfucosylated	Total
RT (min)	11.5–13.3	13.0–15.5	13.5–15.2	12.8–13.8	12.0–13.0	11.1–11.6	H4N2 + H5N2	H5N3S1F1 + H5N3S1	H5N4S2F1 + H5N4S2	Monosialylated + Disialylated	H5N4S2F1 + H5N4S1F1	H4N2 + H5N4S2 + H6N3S1 + H5N2	
[M + 3H]^3+^	720.01	1152.16	1103.47	1055.13	992.77	773.69							
BPH003		4.27		1.62	2.29			3.91	4.27	8.18	5.89	2.29	8.18
BPH004		0.70		2.53	3.58			6.12	0.70	6.81	3.23	3.58	6.81
BPH005		0.13	0.60	0.38	0.50			0.88	0.73	1.61	0.51	1.10	1.61
BPH006		0.71	0.21	0.94	5.95			6.89	0.93	7.81	1.65	6.16	7.81
BPH009		0.21	0.12	0.34	0.01			0.35	0.33	0.68	0.55	0.13	0.68
BPH012	0.14	0.63	0.18	0.89	0.74	0.35	0.49	1.63	0.82	2.45	1.53	1.41	2.94
BPH015	0.07	0.62	0.17	0.50	0.31	0.05	0.12	0.81	0.79	1.59	1.12	0.59	1.71
BPH016		0.38	0.02	0.61	0.17			0.78	0.40	1.18	0.99	0.19	1.18
BPH017	0.59	0.32	0.25	1.29	1.15	0.62	1.21	2.44	0.57	3.01	1.61	2.61	4.22
BPH019	0.68	0.28	0.25	0.10	0.81		0.68	0.91	0.53	1.44	0.38	1.74	2.12
BPH020				0.23	0.55			0.78		0.78	0.23	0.55	0.78
BPH021	0.95	0.31		1.12	0.30	0.29	1.24	1.42	0.31	1.73	1.43	1.54	2.97
BPH022	0.16	0.65	0.42	0.51	0.45	0.15	0.32	0.96	1.07	2.03	1.16	1.19	2.35
BPH025	0.34	0.69	0.60	1.03	0.56		0.34	1.59	1.29	2.89	1.72	1.50	3.23
BPH029	0.53	0.30	0.07	0.48	0.68	0.52	1.05	1.15	0.38	1.53	0.78	1.79	2.58
BPH030		0.50		0.32	0.11			0.43	0.50	0.93	0.81	0.11	0.93
BPH031	0.34	0.24	0.12	0.65	0.44	0.58	0.91	1.10	0.36	1.46	0.89	1.48	2.37
BPH032	0.13	0.40	0.24	0.49	0.30	0.12	0.25	0.80	0.64	1.43	0.89	0.79	1.68
BPH033		0.04		0.06	0.04	0.07	0.07	0.11	0.04	0.14	0.10	0.11	0.21
BPH034	0.09	0.55	0.36	0.50	0.65	0.10	0.18	1.15	0.91	2.06	1.05	1.19	2.25
BPH035	0.24	0.32	0.11	0.49	0.37	0.28	0.52	0.86	0.43	1.30	0.81	1.00	1.82
BPH036		0.06		0.17		0.25	0.25	0.17	0.06	0.23	0.23	0.25	0.48
BPH037	0.35	0.33	0.08	0.34	0.37	0.29	0.63	0.71	0.41	1.12	0.66	1.09	1.75
BPH038	0.75	1.16	0.89	0.44	1.30		0.75	1.74	2.05	3.80	1.60	2.94	4.55
BPH039		0.13		1.37	0.02	0.32	0.32	1.39	0.13	1.52	1.50	0.34	1.84
BPH040	0.78	0.24	0.06	0.14	1.04	0.73	1.51	1.18	0.30	1.48	0.38	2.61	2.98
BPH042				0.52	0.14			0.66		0.66	0.52	0.14	0.66
BPH044	0.24	0.53	0.25	0.49	0.68	0.34	0.58	1.16	0.78	1.95	1.02	1.51	2.53
BPH045		0.79	6.22		2.33			2.33	7.01	9.34	0.79	8.55	9.34
BPH046				0.15	0.13	0.17	0.17	0.29		0.29	0.15	0.30	0.45
BPH047	0.10	0.41	0.35	0.49	0.58	0.10	0.21	1.07	0.76	1.83	0.90	1.13	2.03
BPH048	0.29	0.49	0.14	0.51	0.49	0.23	0.52	1.00	0.64	1.64	1.00	1.16	2.16
BPH049		0.78	0.16	0.58	0.15			0.73	0.94	1.67	1.36	0.31	1.67
BPH050				0.80				0.80		0.80	0.80		0.80
BPH051		1.91		0.96	2.09			3.06	1.91	4.96	2.87	2.09	4.96
BPH054	0.95	0.07	0.06	0.65	0.88	1.17	2.11	1.53	0.13	1.66	0.73	3.05	3.77
BPH055		0.17	0.16	0.29	0.20			0.49	0.32	0.81	0.45	0.36	0.81
BPH057	0.24	0.92	0.09	0.21	0.27	0.64	0.88	0.48	1.00	1.48	1.13	1.23	2.36
BPH058	1.31	0.65	0.07	0.09	1.23	1.18	2.49	1.32	0.72	2.04	0.74	3.79	4.53
BPH060	0.10	0.59	0.19	0.39	0.29	0.06	0.16	0.67	0.78	1.45	0.97	0.64	1.61
BPH061		0.12	0.15	0.33	0.48	0.14	0.14	0.81	0.27	1.08	0.44	0.77	1.22
BPH063		0.57		2.54	2.85			5.39	0.57	5.96	3.11	2.85	5.96
BPH068		0.44	0.11	0.61	0.17	0.08	0.08	0.78	0.54	1.32	1.05	0.35	1.40
PCa131		1.57							1.57	1.57	1.57		1.57
PCa133		0.14	0.06	0.36	0.13			0.49	0.20	0.69	0.50	0.19	0.69
PCa135	0.85	0.09	0.16	0.24	0.34		0.85	0.58	0.25	0.83	0.33	1.35	1.68
PCa137		0.40			1.04			1.04	0.40	1.44	0.40	1.04	1.44
PCa139		0.26	0.12	0.27	0.19			0.46	0.38	0.84	0.53	0.31	0.84
PCa143				0.62				0.62		0.62	0.62		0.62
PCa145		0.49	0.33	0.49	0.30			0.79	0.82	1.61	0.98	0.63	1.61
PCa152		0.65	0.09	0.46	0.59			1.05	0.74	1.79	1.10	0.68	1.79
PCa157		0.13	0.07	0.37	0.17			0.55	0.20	0.75	0.51	0.24	0.75
PCa161	0.25	0.28	0.11	0.29	0.55	0.34	0.59	0.83	0.39	1.22	0.57	1.24	1.81
PCa162	0.17	0.42	0.11	0.28	0.24	0.19	0.36	0.52	0.52	1.04	0.69	0.71	1.40
PCa165					0.47	0.42	0.42	0.47		0.47		0.89	0.89
PCa166	0.98	0.14			0.41	0.28	1.26	0.41	0.14	0.55	0.14	1.68	1.82
PCa171	0.28	0.26	0.02	0.34	0.26	0.41	0.69	0.60	0.28	0.87	0.60	0.97	1.57
PCa003		0.22		0.27	0.71			0.97	0.22	1.20	0.49	0.71	1.20
PCa012		1.99		0.09	0.38			0.47	1.99	2.45	2.07	0.38	2.45
PCa013		1.01	0.09	0.21	0.79			1.00	1.11	2.11	1.22	0.89	2.11
PCa017	0.22	0.44	0.06	0.44	0.28	0.21	0.43	0.71	0.49	1.21	0.87	0.76	1.63
PCa019		0.51		0.21	1.01			1.21	0.51	1.72	0.71	1.01	1.72
PCa025		0.11		0.18	0.64			0.82	0.11	0.93	0.29	0.64	0.93
*p*-value	0.478	0.137	0.137	<0.001^*∗∗∗*^	0.043^*∗*^	0.637	0.513	0.004^*∗∗*^	0.122	0.006^*∗∗*^	0.054	0.003^*∗∗*^	<0.001^*∗∗∗*^

(1) The retention time (RT) of the selected reference peptide (KIDTIVANP, [M + 2H]^2+^ = 485.78) was 16–18 min in 30 min LC running time.

(2) Grouped glycoforms are a combination of the level of indicated glycopeptides in each individual sample. “Total” includes all six selected glycopeptides.

(3) Each sample was analyzed and duplicated and the average is shown.

(4) *∗* indicates *p* < 0.05, *∗∗* indicates *p* < 0.01, and *∗∗∗* indicates *p* < 0.001.

**Table 5:** Areas under the ROC curves of the target PSA glycopeptides.

Glycan composition	AUC^*∗*^	*p* value	Cancer threshold	Sensitivity	Specificity
H5N4S1F1	0.7381 ± 0.0661	0.009	<0.470	92.9%	59.0%
Monosialylated	0.7102 ± 0.0681	0.015	<1.060	100%	47.5%
Sialylated	0.6938 ± 0.0763	0.025	<1.260	68.8%	75%
Unfucosylated	0.6758 ± 0.0699	0.041	<1.065	87.5%	60%
Total	0.7242 ± 0.0662	0.009	<1.815	87.5%	60%

^*∗*^AUC was calculated based on the samples which could be detected for the indicated glycoform or grouped glycoforms.

**Table 6:** Velos Pro validation of samples with low PSA levels.

Sample	ELISA (ng/mL)	Characteristic fragment ions of PSA internal reference peptide (*m*/*z*)			
		856.48	741.45	570.41	571.35
BPH066	<1	+	+	+	+
BPH069	<1	+	+	+	+
PCa010	<1	+	+	+	+
PCa018	<1	+	+	+	+
PCa029	<1	+	+	+	+
PCa030	<1	+	+	+	ND^*∗*^

^*∗*^ND: not detected.

## Abbreviations

PSA: Prostate-specific antigen
PCa: Prostate cancer
BPH: Benign prostate hyperplasia.
